# The Coffee Berry Borer (*Hypothenemus hampei*) Invades Hawaii: Preliminary Investigations on Trap Response and Alternate Hosts

**DOI:** 10.3390/insects3030640

**Published:** 2012-07-11

**Authors:** Russell H. Messing

**Affiliations:** University of Hawaii at Manoa, Kauai Agricultural Research Center, 7370 Kuamoo Rd., Kapaa, HI 96746, USA; E-Mail: messing@hawaii.edu; Tel.: +1-808-822-4984; Fax: +1-808-822-2190

**Keywords:** coffee berry borer, *Hypothenemus hampei*, Hawaii, Kona, trapping

## Abstract

In August 2010 the coffee berry borer, *Hypothenemus hampei*, was first reported to have invaded the Kona coffee growing region of Hawaii, posing a severe economic challenge to the fourth largest agricultural commodity in the State. Despite its long and widespread occurrence throughout the tropics as the most serious pest of coffee, there are still discrepancies in the literature regarding several basic aspects of berry borer biology relevant to its control. In Kona coffee plantations, we investigated the beetles’ response to several trap and lure formulations, and examined the occurrence of beetles in seeds of alternate host plants occurring adjacent to coffee farms. While traps were shown to capture significant numbers of beetles per day, and the occurrence of beetles in alternate hosts was quite rare, the unique situation of coffee culture in Hawaii will make this pest extremely challenging to manage in the Islands.

## 1. Introduction

The coffee berry borer (CBB), *Hypothenemus hampei *(Ferrari), is the most serious insect pest of coffee worldwide, causing severe economic damage in every region where commercial coffee is grown. For many years, Hawaii coffee growers had developed successful horticultural and pest management programs in the absence of this pest. However, the recent invasion of the beetle in coffee farms of the Kona region of the Big Island [1] threatens to seriously impact the entire coffee industry throughout the Hawaiian Islands. This industry comprises approximately 8,000 acres, with total farm revenues of about 30 million dollars, which makes it the fourth largest crop in the State [2]. 

The beetle spends most of its life cycle inside the coffee berry, thus it is particularly difficult to control with chemical or biological inputs [3]. Although the pest has enormous economic impacts and has been intensively studied in many regions around the world, few effective and sustainable control methods have yet to be devised. Endosulfan (an organochlorine insecticide) has been widely used, with some success, but is currently being phased out in the U.S. and elsewhere due to its high mammalian toxicity and environmental damage [4]. Classical biological control with parasitoids has had limited success wherever it has been tried (mostly with African parasitoids transported to Latin America) [5]. However, Hawaii is home to a large number of native Scolytid beetles (closely related to the coffee berry borer), including at least one congener in the genus *Hypothenemus* (*H. ruficeps*), and 19 species in the genus *Xyleborus* [6]. These are potential non-target species of concern for biological control—thus importation and release permits for parasitoids will be exceedingly difficult to obtain in the foreseeable future. The fungal pathogen *Beauveria bassiana*, as well as the fungus *Isaria fumosorosea *and several entomopathogenic nematodes have also been tried in various locations for berry borer control, with only limited effect [7]*. *Cultural control (comprised first and foremost of effective field sanitation to remove remaining infested berries after the harvest) remains the main line of defense against this pest in coffee growing regions throughout the world.

Traps have been used in many countries to monitor flying adult coffee berry borers and, in some cases, to attempt to manage these populations by “mass-trapping”. However, despite their long and widespread use, there are considerable discrepancies in the literature regarding optimal trap design and deployment. Researchers in Brazil [8] and in New Caledonia [9] both found a 1:1 ratio of methanol: ethanol attractive to adult beetles, while others in Brazil [10] found nominally higher CBB captures in traps using a 2:1 or 3:1 rather than a 1:1 ratio. In El Salvador, studies showed marginally higher catches with the 1:1 ratio [11]. In neither of the latter two cases were the capture differences between the ratios statistically significant. Traps with a 3:1 ratio significantly outperformed traps with a 1:1 ratio in Bolivia [12]. Commercial CBB lures sold in the United States use a 3:1 methanol: ethanol ratio (AgBio, Westminster, CO, USA). 

Adult beetles migrate readily across the landscape; so coordinated area-wide or regional control of the beetle is more likely to be successful than individual farm-by-farm efforts. In this context, it is important to determine to what extent berry borers successfully utilize other host plants besides coffee in surrounding ecosystems. Here, again, there are discrepancies in the literature, with several reports listing numerous alternate hosts for adult CBB feeding (if not egg-laying), particularly in the Fabaceae and Rubiaceae [13,14], while other workers [15] report that the beetle is essentially monophagous on coffee.

In this paper we report on initial investigations into coffee berry borer response to traps, and the beetles’ occurrence in alternate host plants in the first year following the CBB invasion of the Kona coffee growing region of Hawaii.

## 2. Methods

### 2.1. Trapping Adult Coffee Berry Borers

**Figure 1:**
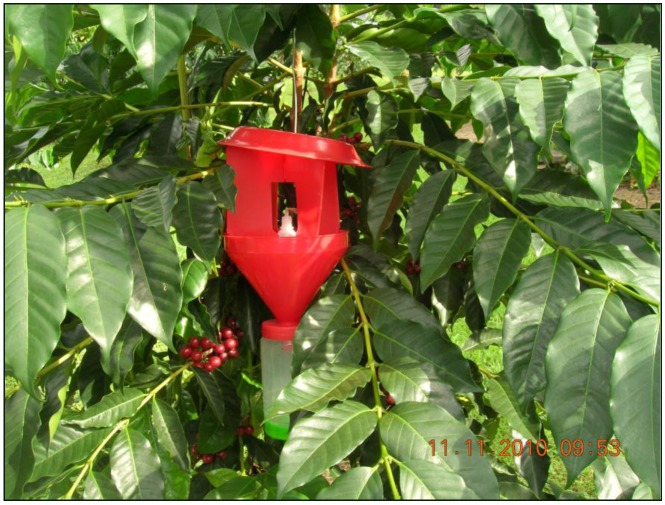
The Brocap trap for the coffee berry borer.

Two cross-baffled red plastic traps (Brocap^®^, obtained from *Agroindustrias Unidas de Mexico*) were placed approximately 12 m apart at each of four sites on a CBB-infested commercial coffee plantation in south Kona, Island of Hawaii (Harrington Farm: N 19°17.648'; W 155°52.036') beginning in mid-November of 2010 (Figure 1). A “standard” beetle attractant used in one of each of the four pairs of traps consisted of a 30 mL vial filled with a 3:1 mixture by volume of methanol: ethanol. Approximately 250 mL of antifreeze (ethylene glycol) was placed in a collection jar at the bottom of the trap to serve as a killing agent and to preserve dead insects. Traps were serviced at weekly to bi-weekly intervals, with fresh attractant and preservative added to the traps, and all insects in the collection jar returned to the laboratory for sorting and identification. 

This set of four standard traps was serviced regularly on the farm for a full year (November 2010–November 2011). Paired with each standard trap, an alternative trap treatment was set in place for four service intervals (a series of four service intervals comprise each separate trial). Four alternatives to the standard trap were tested successively: (1) a 1:1 rather than a 3:1 ratio of methanol: ethanol as the attractant; (2) 100% isopropyl alcohol as the attractant; (3) a 3:1 ratio of methanol: ethanol enclosed in a semi-permeable plastic membrane (Scentry Biologicals, Billings, MT, USA) rather than in a vial as the attractant; (4) a 3:1 ratio of methanol: ethanol in a vial as the attractant, but with one half of a Hercon Vaportape^®^ in each collection jar as the killing agent, rather than ethylene glycol. Thus, overall, there were four separate trials testing alternatives to the standard trap; each trial was replicated four times in succession; and each replicate consisted of four spatially separated pairs of traps. 

All *Hypothenemus* beetles as well as other non-target insects found in the traps were collected, sorted, and counted at each service interval. Counting non-target insects allowed us to have some measure of specificity as well as efficacy in trap capture. As the interval between servicing in each case was not exactly 7 days, all insect counts were divided by the actual number of days each trap was deployed in the field; results are presented as number of insects per trap per day. Comparisons of standard traps with alternative traps in each successive trial were made using Matched Pair Analyses, with service intervals as a blocking factor [16].

Standard traps remained in the field continuously from November 2010–November 2011, thus we also were able to obtain a yearlong overview of the general trend of seasonal phenology of flying CBB adults for this site. To supplement these data, four additional traps of the standard configuration were placed at a second commercial coffee plantation in central Kona (Lehu’ula Farm: N 19°32.028'; W 155°55.749'). These traps were serviced on a weekly basis by a cooperating farmer. In this case, however, counts for each individual trap were not kept separated; rather, the total numbers of insects collected in all four traps were combined. Counts for Lehu’ula Farm therefore have no error term, and are not statistically comparable to the results from Harrington Farm.

### 2.2. Alternate Hosts of Coffee Berry Borer

Within the known area of coffee berry borer infestation on the western (Kona) side of the Big Island of Hawaii, seeds were collected from a number of potential alternate host plants (primarily in the Fabaceae and Rubiaceae) that might serve as reservoirs for beetles during periods of coffee bean scarcity. Besides taxonomy, additional plants were chosen on the basis of accessibility, abundance, proximity to coffee farms, and production of seeds of sufficient size to contain CBB adults. For each plant, seeds were brought to the laboratory, where they were counted or weighed, and half were immediately dissected under a microscope to examine for CBB adults, pupae, or larvae. The other half of the seeds were placed on screens within closed Berlese funnels with a single 40 w light bulb directly over the seeds for ~12 h, at which point any beetles driven by the heat into the collecting jar beneath the funnel were collected and identified. 

## 3. Results and Discussion

### 3.1. Trapping

**Figure 2:**
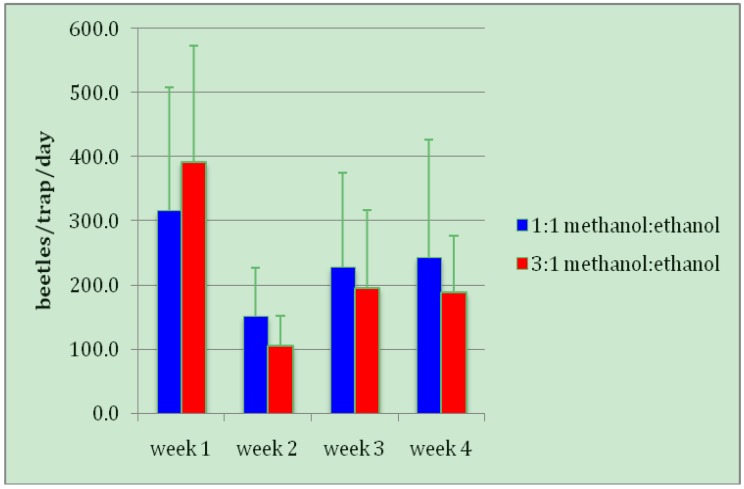
Mean number of adult coffee berry borers captured per trap per day in Brocap traps containing two different ratios of methanol: ethanol.

Brocap traps containing a 3:1 ratio of methanol: ethanol captured a mean (±SE) of 220.6 ± 54.1 adult coffee berry borers per trap per day, which was not significantly different from the 234.9 ± 70.2 beetles caught in traps containing a 1:1 ratio of methanol: ethanol (*t *= 0.4751, *DF *= 15, *p *= 0.6416; Figure 2). This is in agreement with previous findings, which demonstrate that both ratios (as well as a 2:1 ratio) perform equally well in CBB field captures. The small (insignificant) differences observed here and in other papers [9,10] likely reflect differences in beetle populations close to each trap.

**Figure 3:**
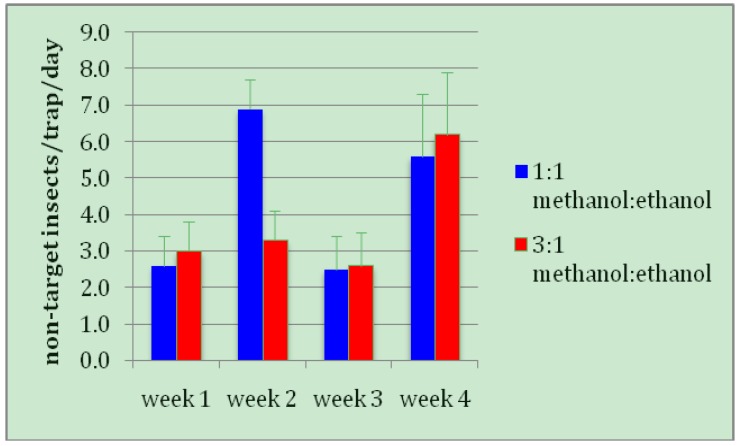
Mean number of non-target adult beetles captured per trap, per day in Brocap traps containing two different ratios of methanol: ethanol.

Methanol: ethanol lures attract other beetle species besides coffee berry borers. Much of the considerable expense of servicing traps on a regular basis is sorting out hundreds to thousands of beetles within each trap, each week, and identifying which are in fact CBB, and which are other non-target beetle species. This discrimination requires each beetle to be individually examined under a dissecting microscope, which can be a lengthy, tedious, and difficult task. For this reason, an alcohol-based lure that is more specific to CBB would be greatly preferable to the standard, even if CBB captures were quantitatively equivalent. However, the results show that 3:1 and 1:1 ratios of methanol: ethanol captured roughly equivalent numbers of non-target insects, predominantly invasive tropical nut borers, (*Hypothenemus obscurus *(F)) and black twig borers, (*Xylosandrus compactus *(Eichhoff)) (Figure 3).

Although during week 2 traps captured twice as many non-target beetles with a 1:1 lure as a 3:1 lure, the overall difference for the four-week trial was not significant (*t *= 0.8420, *DF *= 15, *p *= 0.4130). Thus there is no biological justification to recommend one alcohol ratio over another in terms of either target (CBB) or non-target beetle captures. If trapping is to be used at all on Hawaii coffee farms, the decision of which ratio of alcohols to use is best made on factors such as cost, availability, and storage considerations. Another factor to consider is the relative rate of evaporation of the two alcohols, where potential greater attractiveness of a high evaporation rate must be balanced against lower labor costs (*i.e.*, less-frequent servicing) of traps with lower evaporation rates.

**Figure 4:**
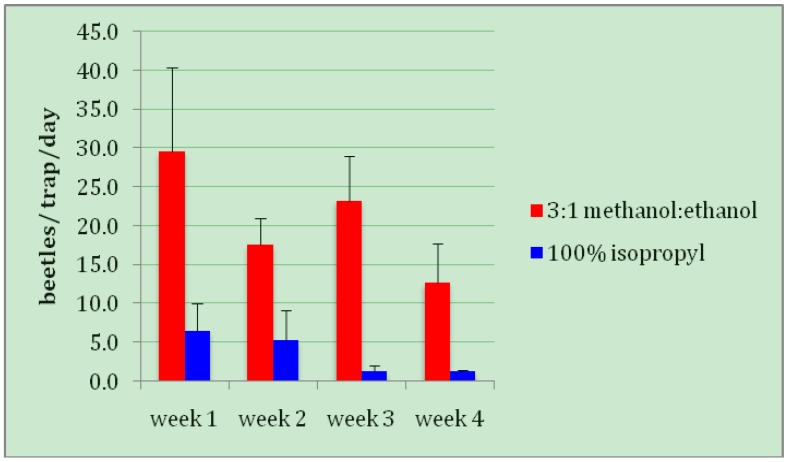
Trap captures of adult coffee berry borers: isopropyl alcohol *vs.* methanol: ethanol mixture.

When CBB trapping first began in Hawaii, within months of its initial discovery, it was noted by farmers and researchers alike that methanol was not widely available in the rural Kona region, especially in small quantities (the majority of coffee farms in Kona are small family farms of just a few acres). The purchase of large (55 gallon; 208 L) drums presented difficulties in storage, and expense. For this reason some questioned whether the far more widely available and less costly isopropyl alcohol might be attractive to the beetles and serve as an alternative lure in CBB traps. However, isopropyl alcohol by itself was far inferior to methanol: ethanol (*t*= 5.1581, *DF *= 16, *p *= 0.0001; Figure 4). To confirm and expand this finding, additional tests would have to include combinations of isopropyl with ethanol, if trapping in fact can be shown to have any role in CBB management in Hawaii in the future.

**Figure 5:**
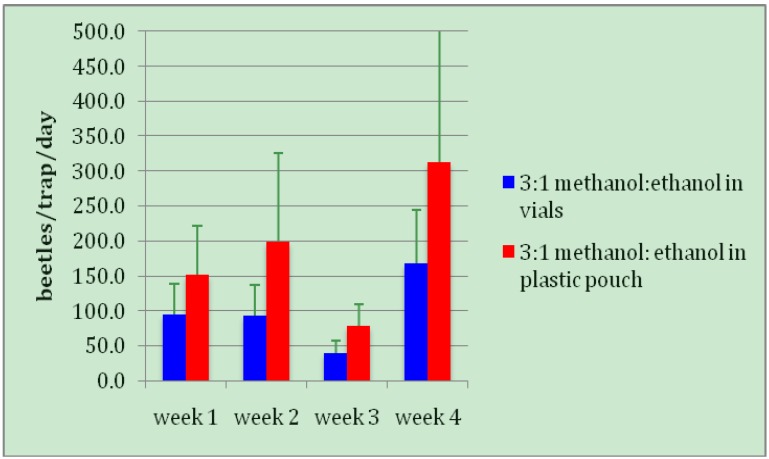
Trap captures of coffee berry borers, using alcohols in vials *vs.* plastic pouches.

One problem of the standard Brocap traps containing vials with methanol: ethanol is the difficulty in regulating the rate of evaporation of the alcohols over the week or longer that traps are left in the field. Hot weather can accelerate the evaporation rate, resulting in the need for more frequent servicing, and hence greater expense. For these reasons we tested traps with a 3:1 methanol: ethanol combination enclosed in a semi-permeable plastic membrane, to determine if it was as effective as traps in which the alcohols evaporate freely from open vials. In fact, traps with the plastic pouches captured significantly more beetles (185.1 ± 55.6) than traps containing open vials of alcohol (98.7 ± 22.9) (*t *= 1.8143, *DF *= 15, *p *= 0.0448; Figure 5). In addition to the improved rate of CBB capture, the pouches are easier to handle and require less frequent servicing than alcohol in vials, and thus would represent cost savings in the labor required to service traps regularly (which must be balanced against the cost of the pouches themselves).

Although ethylene glycol kills beetles and is an effective insect preservative within the traps, it is toxic to humans (especially children) and also to dogs, cats, and other animals. It increases labor costs because it is difficult for workers to handle safely when servicing traps in the field. It provides low volatile toxicity for CBB in the traps. For these reasons we tested the far more easily handled Hercon Vaportapes (one-half strip per trap) to analyze any differences in CBB captures. Vaportapes contain the active ingredient dichlorvos (DDVP), which provides rapid knockdown of beetles in the traps. Standard Brocap traps containing Vaportapes captured 9.2 ± 1.9 beetles/trap/day, not significantly different from the 8.1 ± 1.2 beetles/trap/day caught in traps containing ethylene glycol (*t *= 0.5449, *DF *= 15, *p *= 0.5938). Given the greatly reduced labor costs of using the Vaporstrips, with greater safety and no reduction in beetle captures, the strips are preferable in any possible future trapping efforts.

**Figure 6:**
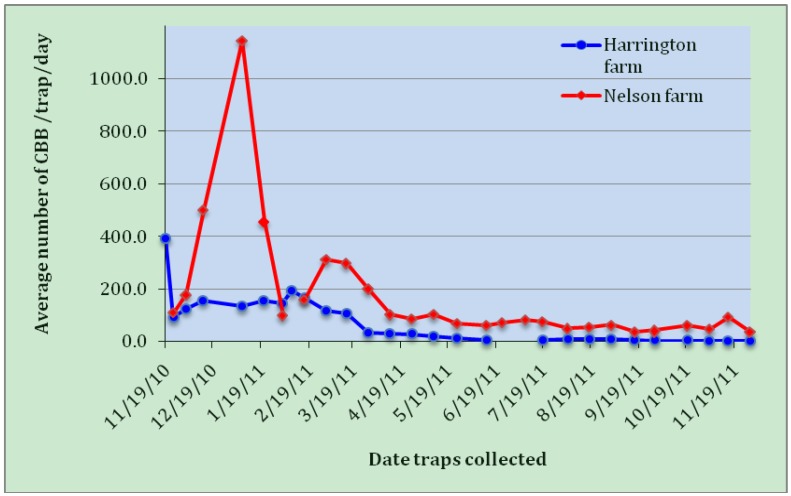
Average number of adult coffee berry borers captured per trap per day on two Kona coffee farms during the first year following coffee berry borer (CBB) invasion in Hawaii.

The overall number of adult coffee berry borers captured (per trap per day) in standard Brocap traps on two Kona coffee farms for a full year is shown in Figure 6. Beetle numbers on the Harrington Farm (south Kona) were at their peak in November 2010, during the height of the coffee harvest, and then decreased steadily until May, after which low numbers were caught for the next six months. On the Nelson farm (central Kona) the numbers peaked in early January, late in the harvest season, and again declined steadily thereafter. It should be noted that the Nelson farm contains a small processing facility for both on-farm and contract coffee berry processing; beetles escaping from this unscreened facility during and immediately after harvest may have inflated the numbers above the natural background level.

Other researchers have also noted highest seasonal trap captures at the time when coffee fruit on the trees declines rapidly [17]. During the off-season (*i.e.*, following the harvest, and before the next year’s crop is sufficiently developed for infestation), low but persistent numbers of CBB captured (some tens per trap per day) indicate a reservoir of adult beetles surviving in coffee berries dropped to the ground, in dried berries remaining on the trees, and in nearby off-farm sources.

### 3.2. Alternate Host Plants

Seeds were collected and examined from 26 different plant species in the Kona region that were thought to be potential alternate hosts for coffee berry borer (Table 1), including 9 species or congeners of species that were previously cited as alternate hosts for CBB reproduction or feeding [14, and references therein]. Only a single plant species in Kona (*Leucanea leucocephala*, or “haole koa”) was found to harbor CBB adults, and for this plant the number of beetles found was exceedingly low (only 3 adult beetles from a total of over 80,000 seeds). There was no visual evidence of reproduction of beetles within these seeds, nor was there evidence of adult feeding (*i.e.*, no eggs or larvae; no frass). These results stand in contrast to those reported in earlier review papers [14], where feeding and even reproduction in alternate hosts was reported from several countries. The reasons for this discrepancy remain unclear. During much of our sampling of potential alternate host plants, coffee berries remained available to some extent in Kona (as irregular rains lead to asynchronous fruiting; and feral coffee plants are not pruned). Alternate host use may be tightly restricted to almost complete absence of the preferred host, temporally or spatially, at scales yet to be determined. Also, it has recently been determined using molecular markers that the nominal species “*Hypothenemus hampei*” occurring throughout the world may in fact represent a complex of cryptic species, or at least distinguishable genetic entities [18]. We cannot rule out the possibility that these geographically and genetically distinct populations may differ to some degree in measurable biological traits, such as the ability to obtain some level of nutrition from alternate seeds during periods of coffee scarcity. We do not yet know the geographic origin of the CBB propagules that were invasive in Hawaii. This is an area that deserves closer scrutiny.

**Table 1:** Potential alternate host plant seeds examined for presence of coffee berry borer by dissection and by Berlese funnel extraction.

Host Plant	Plant family	Common name	Amount of seed (grams)	CBB dissected	CBB in Berlese
*Cajanus cajan*	Fabaceae	pigeon pea	301.6	0	0
*Cesalpenia pulcherrima*	Fabaceae	dwarf poinciana	65.0	0	0
*Chamaecrista nictitans*	Fabaceae	partridge pea	52.0	0	0
*Crotalaria incana*	Fabaceae	shake shake	334.8	0	0
*Crotalaria pallida*	Fabaceae	smooth rattle pod	12.1	0	0
*Crotolaria *sp*. *	Fabaceae	rattle pod	26.0	0	0
*Delonix regia*	Fabaceae	flame tree	96.2	0	0
*Desmodium intortum*	Fabaceae	tick trefoil	8.4	0	0
*Desmodium *sp*.*	Fabaceae		88.3	0	0
*Desmodium tortuosum*	Fabaceae	Florida beggarweed	43.0	0	0
*Erythrina x bidwilli*	Fabaceae	coral tree	158.4	0	0
*Eugenia uniflora*	Myrtaceae	Surinam cherry	753.2	0	0
*Euphorbia cyathophora*	Euphorbiaceae	wild poinsettia	41 seeds	0	0
*Euphorbia heterophylla*	Euphorbiaceae	Mexican fireplant	16.8	0	0
*Indigofera suffruticosa*	Fabaceae	wild indigo	104.9	0	0
*Ixora *sp*.*	Rubiaceae	ixora	16.0	0	0
*Leucanea leucocephala*	Fabaceae	haole koa	2,528.5	0	**3**
*Passiflora edulis*	Passifloraceae	lilikoi	403.4	0	0
*Passiflora tarminiana*	Passifloraceae	banana poka	78.6	0	0
*Pentas lanceolata*	Rubiaceae	star flower	9.4	0	0
*Pritchardia *sp*.*	Arecaceae	loulou palm	392.5	0	0
*Schefflera arboricola*	Araliaceae	dwarf schefflera	196.8	0	0
*Schinus terebinthefolius*	Anacardiaceae	Christmas berry	5.2	0	0
*Senna *sp.	Fabaceae		173.0	0	0
*Strongylodon macrobotrys*	Fabaceae	jade vine	122.1	0	0
*Vigna speciosa*	Fabaceae	wandering cowpea	41.0	0	0

Even though the infestation level of CBB in haole koa seeds was very low, it might be argued that the high abundance and broad distribution of this weed throughout the Kona region could lead to a significant area-wide beetle reservoir. However, there are several arguments tempering this suggestion. First, the production of coffee fruit (which can harbor up to a hundred beetles per seed) is not completely synchronized on coffee farms throughout the region. Even though there is a distinct harvest season during autumn and early winter months, coffee fruits are available to some extent throughout the year (depending on farm elevation, rainfall patterns, *etc.*). Thus the significance of the very minor haole koa refuge compared to coffee fruit itself on the farms is diminished. Second, there is an enormous known reservoir of coffee berry borers in the widespread (fruiting) feral coffee plants that have been spread by birds, rats, and pigs throughout ravines and other unmanaged lands prevalent in the Kona region. This feral coffee, along with a substantial number of unmanaged or abandoned coffee plantations in the region, represents a huge source of beetles that will continuously re-infest even the most tightly managed farms, and is the biggest single obstacle to implementing IPM strategies against CBB in Hawaii.

Coffee is the second most widely traded commodity in the world (after petroleum products). It is grown in over 80 countries, and contributes to the livelihood of tens of millions of farmers, all of whom face potential damage from the coffee berry borer. Coffee culture in Hawaii, however, is unique in several important aspects, which will require regionally specific approaches to management of this challenging pest.

While traps using a mixture of ethanol and methanol capture significant numbers of CBB, it is unclear what role traps might play in integrated control of this pest in Hawaii. When viewed in the context of helping Kona’s coffee farmers mitigate CBB damage and economic loss, it is difficult to see a useful role for traps unless more powerful lures can be discovered. Trapping is recognized to be of value in monitoring presence or absence of coffee berry borer, but to date limited research has documented quantitative correlations between trap catch and damage to growers’ fields (though see [19]). In areas such as the Kona region of Hawaii, where the beetle has spread very rapidly and now occurs on virtually every farm, this limits the usefulness of trapping to specialized research objectives, or perhaps to obtain only a general sense of the population density in an area. Even were action thresholds based on trap counts to be accurately determined, there are no effective mitigation responses available to quickly reduce beetle populations before Economic Injury Levels are reached.

There are limited published data available in the grey literature to support “mass-trapping” as a tool to reduce farm level populations of CBB. Although traps may capture thousands of beetles per day, this is generally a small percentage of the total CBB population. For example, workers in Columbia found as many as 3 million beetles per acre in coffee berries that were not removed before pruning [20]. The promising mass-trapping results reported by some [21,22] occurred in commercial coffee production areas of El Salvador where sanitation was part of the standard background cultural control practice, greatly minimizing baseline beetle population pressure. Kona’s coffee agro-ecosystems will face great difficulty in reducing this baseline, for three main reasons: (1) most of the farms are small, pest management tactics are not well coordinated farm-to-farm, and there are significant numbers of neglected or abandoned farms in the region; (2) there is a great deal of wild (feral) coffee in the area that serves as a reservoir to re-infest managed farms; and (3) farm labor costs in Hawaii are among the highest in the world (Table 2); this makes the labor-intensive sanitation efforts necessary to remove infested berries from the ground and trees following harvest cost prohibitive to the majority of growers in the region.

**Table 2:** High cost of farm wages in Hawaii compared to other regions monthly wage in U.S. dollars. Reprinted with permission from [23].

Rank	Country	1998	1999	2000	2001	2002	2003	2004	2005	2006	2007	2008	Average
1	Hawaii				1,856	1,904	1,944	1,932	2,060	2,180	2,276	2,348	2,062
	U.S.	1,260	1,360	1,388	1,484	1,496	1,588		1,608	1,688	1,648	1,776	1,530
2	Australia	1,101	1,210	1,090									1,134
3	Italy												799
4	Costa Rica	198	223	216	206			237	218			199	214
5	Brazil	274	183	183	162	140							188
6	Mexico	114	127	149	180	184	181	183	204	215	228	239	182
7	Columbia					62	80	166	114	137	156		119
8	Philippines	90	112		107		116		133				111
9	Thailand				47	131	57			81	95		82

**Notes:** Published data are in local currency units. Conversion to US dollars used published exchange rate from the World Bank. **Sources:** Country data are from [24]. Data for Italy are from [25]. Hawaii data are from the 2008 Employment and Payrolls in Hawaii.

While the challenges of CBB management under Hawaii conditions are acute, there are several possibilities for improved control that remain to be explored. New chemical insecticides are continuously being developed and tested. Novel, perhaps mechanized techniques to remove post-harvest berries from the trees and from the ground could help prevent continuous re-infestion within individual farms; while tall hedgerows might help to limit in-migration of this weak-flying beetle. Semiochemicals based on coffee berry volatiles might be identified, synthesized, and used to improve trap performance. Genetic constructs carrying CRY 1 genes from beetle-pathogenic strains of *Baccilus thuringiensis* might be engineered into the coffee genome. Kaolin clay particles, neem oil, and other essential oils may help to deter beetle host-finding and oviposition. Combinations of separate strains of *Beauveria bassiana* [26] may provide a synergistic effect to enhance the pathogen’s efficacy as an inundative biocontrol agent. Each of these techniques faces its own obstacles and challenges, thus the ingenuity and determination of growers and researchers alike will be called upon over the next few years to save this important local industry.

## 4. Conclusions

Red Brocap traps containing either a 1:1 or 3:1 ratio of methanol: ethanol, with Hercon Vaportapes as the killing agent, can capture hundreds up to thousands of adult female coffee berry borers per day in Kona coffee plantations. Very small numbers of CBB can be found in the seeds of a common weed plant in the area, haole koa (*Leucanea leucocephala*), with no evidence of reproduction or feeding. The importance and utility of trapping, and the significance of the low number of beetles in an alternate host, are much less in Hawaii than in other coffee-growing regions due to the huge reservoir of beetles in feral and abandoned coffee, and the high cost of labor that would be required to perform intensive sanitation. The coffee berry borer will pose an enormous economic challenge for the coffee farmers of Hawaii unless novel control tactics can be devised.

## References

[1] Burbano E.G., Wright M.G., Bright D.E., Vega F. (2011). New record for the coffee berry borer, *Hypothenemus hampei*, in Hawaii. J. Insect Sci..

[2] USDA. NASS (2010). Statistics of Hawaii Agriculture for 2008. http://www.nass.usda.gov/Statistics_by_State/Hawaii/Publications/Annual_Statistical_Bulletin/all2008.pdf.

[3] Jaramillo J., Borgemeister C., Baker P.S. (2006). Coffee berry borer *Hypothenemus hampei* (Coleoptera: Curculionidae): Searching for sustainable control strategies. Bull. Entomol. Res..

[4] US EPA (2010). Endosulfan phase out. http://www.epa.gov/pesticides/reregistration/endosulfan/endosulfan-agreement.html/.

[5] Jaramillo J., Bustillo A.E., Montoya E.C., Borgemeister C. (2005). Biological control of the coffee berry borer *Hypothenemus hampei* (Ferrari) (Coleoptera: Curculionidae, Scolytinae) by *Phymastichus coffea* LaSalle (Hymenoptera: Eulophidae) in Colombia. Bull. Entomol. Res..

[6] Bishop Museum (2012). Hawaiian Terrestrial Arthropod Checklist. http://www2.bishopmuseum.org/HBS/checklist/query.asp?grp=Arthropod/.

[7] Vega F.E., Infante F., Castillo A., Jaramillo J. (2009). The coffee berry borer *Hypothenemus hampei* (Ferrari) (Coleoptera: Curculionidae): A short review, with recent findings and future research directions. Terr. Arthropod Rev..

[8] Mendoza-Mora J.R. (1991). Resposta da Broca-do-cafe, *Hypothenemus hampei*, a Estimulos Visuais e Semioquimicos.

[9] Mathieu F., Brun L.O., Marcillaud C., Frérot B. (1997). Trapping of the coffee berry borer *Hypothenemus hampei* Ferr. (Col. Scolytidae) within a mesh-enclosed environment: Interaction of olfactory and visual stimuli. J. Appl. Entomol..

[10] da Silva F.C., Ventura M.U., Morales L. (2006). Capture of *Hypothenemus hampei* Ferrari (Coleoptera: Scolytidae) in response to trap characteristics. Sci. Agric. (Piracicaba Braz.).

[11] Dufour B., Frérot B. (2008). Optimization of coffee berry borer, *Hypothenemus hampei* Ferrari (Col: Scolytidae), mass trapping with an attractant mixture. J. Appl. Entomol..

[12] Agramont R., Cuba N., Beltran J.L., Almanza J.C., Loza-Murguia M. (2010). Crafting traps with attractant alcoholics an alternative for monitoring and control of borer coffee, *Hypothenemus hampei* (Ferrari 1867). J. Selva Andina Res. Soc..

[13] Vijayalakshmi C.K., Abdul Rehiman P., Reddy A.G.S. (1994). A note on the alternative shelters of coffee berry borer beetles. J. Coffee Res..

[14] Damon A. (2000). A review of the biology and control of the coffee berry borer, *Hypothenemus hampei* (Coleoptera: Scolytidae). Bull. Entomol. Res..

[15] Johanneson N.E., Mansingh A. (1984). Host pest relationship of the genus *Hypothenemus* (Scolytidae: Coleoptera) with special reference to the coffee berry borer (*H. hampei*). J. Coffee Res..

[16] SAS Institute (2010). JMP® Pro Statistical Software, (version 9.0.2).

[17] Mathieu F, Brun L.O., Frérot B., Suckling D.M., Frampton C. (1999). Progression in field infestation is linked with trapping of coffee berry borer, *Hypothenemus hampei* (Col., Scolytidae). J. Appl. Entomol..

[18] Gauthier N. (2010). Multiple cryptic genetic units in *Hypothenemus hampei* (Coleoptera: Scolytinae): evidence from microsatellite and mitochondrial DNA sequence data. Biol. J. Linnean Soc..

[19] Pereira A.E., Vilela E.F., Tinoco R.S., de Lima J.O., Fantine A.K., Morais E.G.F., Franç C.F.M. (2012). Correlation between numbers captured and infestation levels of the Coffee Berry-borer, *Hypothenemus hampei*: A preliminary basis for an action threshold using baited traps. Int. J. Pest Manag..

[20] Bustillo A.E., Cardenas R., Villalba D., Benavides P., Orozco J., Posada F. (1998). Manejo integrado de la broca del café *Hypothenemus hampei* (Ferrari) en Colombia.

[21] Dufour B.P., González M.O., Frérot B. Mass Trapping of Coffee Berry Borer *Hypothenemus hampei* Ferr. (Coleoptera: Scolytidae) under Field Conditions: First Results. Proceedings of the Dix-huitieme Colloque Scientifique International sur le Café.

[22] Dufour B.P., González M.O., Mauricio J.J., Chávez B.A., Ramírez R. Validation of Coffee Berry Borer Trapping with Brocap Trap. Proceedings of the 20-eme Colloque Scientifique International sur le Café.

[23] Parcon H., Arita S., Loke M., Leung P. (2011). A comparison of agricultural input prices: Hawaii *vs.* its major export competitors. Economic Issues EI-20.

[24] LABORSTA Internet. http://laborsta.ilo.org/.

[25] Il sistema informativo sul lavoro online agri-info. http://agri-info.eu/italiano/a_start.php/.

[26] Cruz L.P., Galan A.L., Gongora C.E. (2006). Exploiting the genetic diversity of *Beauveria bassiana* for improving the biological control of the coffee berry borer through the use of strain mixtures. Appl. Microbiol. Biotech..

